# Increasing access to CBT for psychosis patients: study protocol for a randomised controlled trial evaluating brief, targeted CBT for distressing voices delivered by assistant psychologists (GiVE3)

**DOI:** 10.1186/s13063-023-07611-7

**Published:** 2023-09-15

**Authors:** Mark Hayward, Katherine Berry, Stephen Bremner, Kate Cavanagh, Guy Dodgson, David Fowler, Heather Gage, Kathryn Greenwood, Cassie Hazell, Anna-Marie Bibby-Jones, Sam Robertson, Morro Touray, Natalie Dailey, Clara Strauss

**Affiliations:** 1 School of Psychology, University of Sussex, Brighton, BN1 9RH UK; 2 Research and Development Department, Sussex Partnership NHS Foundation Trust, Hove, BN3 7HZ UK; 3 Faculty of Biology, University of Manchester, Medicine & Health, Manchester, M13 9PL UK; 4 Brighton & Sussex Medical School, University of Sussex, Brighton, BN1 9RH UK; 5 Department of Psychology, University of Durham, Durham, DH1 3LE UK; 6 School of Biosciences & Medicine, University of Surrey, Guildford, GU2 7XH UK; 7 School of Psychology, University of Surrey, Guildford, GU2 7HX UK

**Keywords:** Self-help, Voice hearing, Psychosis, CBT, Randomised controlled trial

## Abstract

**Background:**

The National Institute for Health and Care Excellence (NICE) recommends that cognitive behaviour therapy (CBT) is offered to all patients with a psychosis diagnosis. However, only a minority of psychosis patients in England and Wales are offered CBT. This is attributable, in part, to the resource-intensive nature of CBT. One response to this problem has been the development of CBT in brief formats that are targeted at a single symptom and are deliverable by briefly trained therapists. We have developed Guided self-help CBT (the GiVE intervention) as a brief form of CBT for distressing voices and reported evidence for the feasibility of a randomised controlled trial (RCT) when the intervention was delivered by briefly trained therapists (assistant psychologists). This study will investigate the clinical and cost-effectiveness of the GiVE intervention when delivered by assistant psychologists following a brief training.

**Methods:**

This study is a pragmatic, two-arm, parallel group, superiority RCT comparing the GiVE intervention (delivered by assistant psychologists) and treatment as usual to treatment as usual alone, recruiting across three sites, using 1:1 allocation and blind post-treatment and follow-up assessments. A nested qualitative study will develop a model for implementation.

**Discussion:**

If the GiVE intervention is found to be effective when delivered by assistant psychologists, this intervention could significantly contribute to increasing access to evidence-based psychological interventions for psychosis patients. Furthermore, implementation across secondary care services within the UK’s National Health Service may pave the way for other symptom-specific and less resource-intensive CBT-informed interventions for psychosis patients to be developed and evaluated.

**Trial registration:**

Current Controlled Trials ISRCTN registration number: 12748453. Registered on 28 September 2022.

## Introduction

### Background and rationale {6a}

Approximately 300,000 people in England and Wales have a diagnosis of psychosis [1]. The symptoms of psychosis such as paranoid delusions and hearing voices (hereafter referred to as distressing voices) cause significant distress and disability and contribute to an increased risk of physical health problems and early mortality [2], and suicide rates up to twelve times greater than the general population [3]. The overall annual societal cost of psychosis in England has been estimated at £11.8 billion [4].

Cognitive behaviour therapy for psychosis (CBTp) has robust evidence for clinical [5] and cost-effectiveness [6] and is recommended by the National Institute for Health and Care Excellence (NICE) to promote the recovery of psychosis patients [6]. However, implementation in the UK is extremely poor, [7] with only 26% of psychosis patients being offered CBTp [1]. One reason for this lack of access is limited resources, [8] as CBTp is resource-intensive in relation to its duration (minimum of 16 sessions recommended by NICE) and delivery (by highly trained therapists—who are trained over a 2–3-year period).

NICE recommend research into two issues that could potentially reduce the resources required to deliver CBTp to psychosis patients [6]. Firstly, the duration of CBTp—how many sessions are required to generate sufficient benefit (research recommendation 9.4.11.1)? Our meta-analysis of 10 controlled studies indicated that CBTp delivered over less than the recommended 16 sessions is effective in reducing psychosis symptoms when delivered by highly trained therapists [9]. This focus upon brief forms of CBTp has recently been taken forward through the development of single-symptom therapies [10] which target only one specific psychotic symptom, with some promising early results [11]. Secondly, the ability of “briefly trained” practitioners to deliver CBTp—can CBTp be delivered by a more cost-effective alternative workforce (i.e., not highly trained therapists) (research recommendation 9.4.11.2)? Evidence suggests that frontline practitioners with case management responsibilities can struggle to fulfil this role as outcomes from trials have been inconclusive [12, 13] and there are competing demands upon their time [14, 15]. Psychology graduates, however, are well-positioned to undertake such a role [14–16] as there is a large number of psychology graduates in the UK; their degree provides them with training in psychological models of emotion and behaviour; they can be readily employed in assistant psychologist (AP) roles at lower cost than highly trained therapists; and they do not have case management responsibilities.

We have packaged CBTp in a brief and structured form that is targeted at the specific psychotic symptom of distressing voices. We collaborated with people with lived experience of psychosis and/or distressing voices to turn our self-help CBT book (Overcoming Distressing Voices [17]) into a workbook (An Introduction to Self-help for Distressing Voices [18]) and created the Guided self-help CBT intervention for distressing VoicEs (the GiVE intervention). We initially evaluated the GiVE intervention when delivered by highly trained therapists, as this allowed the evaluation to focus exclusively upon CBT packaged in this form, rather than any interaction between the GiVE intervention and delivery by briefly trained therapists. The findings from a pilot randomised controlled trial (RCT) of GiVE compared to usual care (*N* = 28) were encouraging with high levels of retention, and a very large between-group effect for the predetermined primary outcome of negative voice-impact (Cohen’s *d* = 1.78) [19]. Our next step was to evaluate the GiVE intervention when delivered by a cost-effective and widely available workforce of APs. We conducted a three-arm, multi-site feasibility RCT comparing the GiVE intervention (delivered by APs over 8 sessions) to a Supportive Counselling intervention (delivered by the same APs over 8 sessions—the active control intervention) and to treatment-as-usual (TAU). The progression criteria were met and suggested that clinicians were willing to refer psychosis patients to the trial, that patients could be retained within the trial at post-treatment and that the APs could adhere to therapy and supervision protocols. A signal of efficacy favouring the GiVE intervention in comparison to both the Supportive Counselling intervention (Cohen’s *d* = 0.77) and TAU (Cohen’s *d* = 0.49) was evident at post-treatment (16 weeks after randomisation) for the candidate primary outcome (voice-related distress) [20]. The lessons learnt from the feasibility RCT have informed the design of the current study.

If the GiVE intervention is found to be clinically and cost-effective in the current study when delivered by APs, it could substantially increase the number of psychosis patients that are able to access and engage with the principles of CBT, which will generate benefits for psychosis patients and the NHS.

### Objectives {7}

The long-term aim of this research is to increase access to CBTp for psychosis patients; this will be achieved by evaluating the clinical and cost-effectiveness of a brief and targeted CBT-informed intervention that can be delivered by a less costly and widely available workforce of APs following a brief training in the delivery of the intervention and ongoing supervision. CBT-informed interventions offered to psychosis patients in less resource-intensive forms as part of an evidence-based intervention pathway will generate benefits for (1) individual patients (reduced distress and enhanced recovery); (2) service-level patient benefit (increased access to evidence-based psychological therapies); and (3) economic benefits to the NHS (in terms of the reduced use of high-cost mental health services).

The proposed study will evaluate the following hypothesis: In comparison to usual care, is the GiVE intervention effective at treating distressing voices when delivered to psychosis patients by briefly trained APs?

### Trial design {8}

This study is a pragmatic, two-arm, parallel group, superiority RCT comparing the GiVE intervention (delivered by APs) in addition to treatment as usual to treatment as usual alone, recruiting across three sites, using 1:1 allocation and blind post-treatment and follow-up assessments.

## Methods: participants, interventions and outcomes

### Study setting {9}

The study will be conducted within Secondary Care Adult Mental Health Services in three large NHS Mental Health Trusts in Sussex (rural), Greater Manchester (urban) and North-East England (rural). The study intervention will be delivered by six therapists (two per site) who will each have their own caseload of 10–11 participants (across the duration of the intervention delivery period).

### Eligibility criteria {10}

1. In contact with Secondary Care Mental Health Services (under the care of a mental health team within one of the recruiting Trusts)
2. Have a clinician-reported diagnosis of psychosis (including schizophrenia spectrum disorder [ICD10 F20–29] or affective disorder with psychotic symptoms [ICD-10 F30–39, subcategories with psychotic symptoms])
3. Aged 18 or over
4. Willing to provide informed consent
5. Experiencing current voice hearing; this will be operationalised by participants having a score of at least 1 on item 1 (‘Frequently’) on the Psychotic Symptoms Rating Scale—Auditory Hallucinations Scale (PSYRATS-AH [21]) at the time of consent—indicating that the participant has experienced at least one episode of voice hearing in the past week
6. Scoring 3 or 4 (rated on a 0–4 scale) on either the intensity of distress item or the amount of distress item on PSYRATS-AH [21] at the time of consent.

#### Exclusion criteria

1. Established organic cause for distressing voices
2. Primary diagnosis of substance misuse
3. Currently detained in hospital under a section of the Mental Health Act
4. Having completed a full course (minimum of 16 h) of CBTp for psychotic symptoms during the past year
5. Immediate and serious risk to self or others (assessed at the point of referral/eligibility review)

If inclusion and exclusion criteria are met, every effort will be made to include and support the participation of patients who experience challenges with respect to language and literacy. This will include the use of the translation and literacy services that are routinely available within the clinical services of the host sites and the informal support that is available within the participant’s network. The recruitment resources will be available in Easy Read versions.

### Who will take informed consent? {26a}

Consent to take part in this study will be informed. All participants will be given the participant information sheet (PIS) at least 24 h before meeting with a research assistant (RA) to give consent. Furthermore, participants will have the opportunity to ask questions about the research study before providing consent. The combination of the PIS and the opportunity to ask questions of the RA will ensure that any consent given will be fully informed.

### Additional consent provisions for collection and use of participant data and biological specimens {26b}

On the consent form, participants will be informed that their data will be retained should they choose to withdraw from the trial. Participants will also be asked for permission for the research team to share relevant data with people from the NHS Trusts taking part in the research or from regulatory authorities, where relevant. This trial does not involve collecting biological specimens for storage.

## Interventions

### Explanation for the choice of comparators {6b}

A two-arm RCT was chosen to enable (1) any effect of GiVE to be differentiated from the impact of standard care (available within TAU); and (2) an evaluation of the cost-effectiveness of GiVE—generated by a comparison to the treatments that are usually offered to patients (available within TAU).

### Intervention description {11a}

#### Guided self-help intervention for voices (GiVE)

The intervention is described in our published workbook [18] and will consist of 10 one-hour sessions of the GiVE intervention over a 16-week period, delivered by APs in NHS clinics, participants’ own homes or other community locations as preferred and appropriate. Where participants express a preference for the intervention to be delivered remotely (by videocall or phone), this preference will be honoured. GiVE is a psychological intervention that targets the mechanisms that have been empirically found to maintain voice-related distress—negative beliefs about voices, [22, 23] negative beliefs about self [24, 25] and negative relating [26, 27]. After the introductory sessions (including a focus upon coping), the intervention will cover three core modules: (1) beliefs about the self, (2) beliefs about voices and (3) relationships. Modules (1) and (2) draw upon psychoeducation and cognitive behavioural strategies to help participants to re-evaluate the accuracy of their negative or unhelpful beliefs related to the self and voices. Module (3) additionally involves work on how to relate to others and voices more assertively.

A participant will have received sufficient ‘exposure’ to the intervention if they have attended a minimum of 6 of the 10 sessions.

Participants will be provided with a copy of the workbook [18] and companion self-help book [17] and will be ‘guided’ through the structured content of the workbook by an AP—with encouragement to complete between-session exercises in the workbook and to read relevant chapters of the self-help book. Participants will also have the opportunity to access the ‘CHOICES’ mobile phone application. The ‘CHOICES’ app was developed by Sussex Partnership NHS Foundation Trust to support the learning of patients within its clinical services. The app has been revised for use in this trial and aims to support the continued use of strategies that have been learnt through the GiVE intervention. If participants have a smart/ iPhone and are familiar with accessing mobile phone apps, the AP will provide them with a guide to access the CHOICES app. The research team will have no access to the data entered by participants into the app.

Consistent with the previous recommendations from Experts-by-Experience (EBEs), we will recruit APs who have some experience of working as an AP with patients with complex needs. The APs will receive a brief 3-day training in the delivery of the GiVE intervention facilitated by the Chief Investigator. The training will include 1 introductory day (covering the experience of distressing voices and CBT principles) and 2 days on the GiVE intervention (covering the content of the workbook) and will be delivered to all APs at the same time.

Two APs will offer the GiVE intervention at each site. The APs will be offered weekly clinical supervision by the Site Leads, including both 1:1 supervision at site and group supervision at both site and remotely across sites. Participants in the GiVE arm will continue to receive treatment as usual (described below) throughout their participation in the study.

#### Treatment-as-usual

Treatment as usual (TAU) will be delivered according to national and local service protocols and guidelines and mainly consist of antipsychotic medication and support and monitoring from the local clinical team, with individual and family psychological therapies offered occasionally.

### Criteria for discontinuing or modifying allocated interventions {11b}

The intervention may be discontinued if the participant requests this or if the AP judges, in consultation with the wider supervisory team, that the intervention is associated with a significant worsening of mental health. In this case, an adverse event form would also be completed. It will be made clear to each participant that, should they find any aspect of the research distressing, including the intervention, and/or no longer wish to continue, they will be able to withdraw without this impacting on their usual clinical care in any way.

### Strategies to improve adherence to interventions {11c}

Therapeutic drift will be minimised by the structure and detail of the workbook and close supervision of the APs by the Site Leads. Adherence to the workbook will be assessed through APs completing a session checklist at the conclusion of each session. All therapy sessions will be audio-recorded (with the participant’s consent—which will be verbally re-affirmed at the beginning of each therapy session) and a random 10% sample rated for adherence and competence by an independent CBT expert. These recordings will be rated for adherence using the session checklist. In addition to providing an independent assessment of the AP’s delivery of the session content, cross-referencing of the self-report and independently rated checklists will offer insights about the ability of APs to monitor their own performance. Session recordings will also be rated for competence using a version of the Cognitive Therapy Scale-Revised (CTS-R) [28] that has been adapted for use within the training of ‘lower-intensity’ therapists within the NHS Talking Therapies services within Sussex.

### Relevant concomitant care permitted or prohibited during the trial {11d}

Participants in both arms of the trial will be encouraged to engage in and continue with existing treatments. Our methodological approach will be to carefully monitor and capture the service contacts received across a range of services in both arms of the trial using an adapted version of the Client Service Receipt Inventory (CSRI) [29].

### Provisions for post-trial care {30}

There is no provision for post-trial care in the study, and participants will remain under the care of their usual mental health services.

### Outcomes {12}

#### Screening measures

1. Psychotic Symptoms Rating Scale—Auditory Hallucinations Scale (PSYRATS-AH) [21]
  This 11-item observer rating scale assesses the characteristics of voices across four scales—distress, attribution, loudness and frequency
  The measure is used at screening to assess the presence of voice hearing experiences and associated levels of distress

#### Demographic measures

2. Information About You survey

#### Clinical measures

3. Primary outcome: Psychotic Symptoms Rating Scale – Auditory Hallucinations (PSYRATS-AH) [21]
  ◦ The 5-item ‘distress’ subscale measures the impact that voices have on the individual and will be the primary outcome
  ◦ The subscale is an observer rated measure that consists of 5 items
  ◦ This measure is included as the study intervention aims to reduce the distress associated with hearing voices
  ◦ The primary endpoint will be 16 weeks post-randomisation.

4. Secondary (clinical) outcome: Psychotic Symptoms Rating Scale—Auditory Hallucinations (PSYRATS-AH) [21]
  ◦ The subscales of attribution (2 items), loudness (1 item) and frequency (3 items) measure the characteristics of voices beyond distress.
  ◦ These characteristics are not targeted by the intervention but can change during therapy.

5. Secondary (clinical) outcome: The Hospital Anxiety and Depression Scale (HADS) [30]
  ◦ Any changes in distress related to voice hearing experience may reflect a more global reduction in emotional distress—therefore this measure will allow such changes to be captured.

6. Secondary (clinical) outcome: CHoice of Outcome In Cbt for psychosEs (CHOICE) [31]
  ◦ The CHOICE was developed in partnership with patients to measure recovery and therefore reflects the outcomes that are important to them
  ◦ The 12-item short form of this measure will be used.

7. Secondary (mechanism) outcome: The Brief Core Schema Scale (BCSS)—self scale [32]
  ◦ One of the modules within the GiVE intervention focuses on beliefs about the self. This 12-item measure assesses the degree of positive and negative beliefs the person has about themselves

8. Secondary (mechanism) outcome: Approve – Voices [33]
  One of the modules within the GiVE intervention focusses on relationships. This 15-item measure assesses the styles of relating to voices across three subscales—assertive, aggressive and passive.

9. Secondary (mechanism) outcome: Approve—Social [33]
  One of the modules within the GiVE intervention focusses on relationships. This 15-item measure assesses the styles of relating to other people across three subscales—assertive, aggressive and passive.

10. Secondary (mechanism) outcome: Beliefs about voices questionnaire—revised (BAVQ-R) [34]
  ◦ One of the modules within GiVE targets beliefs about voices. This measure can assess the degree to which these beliefs are positive or negative, and may be changing during therapy
  ◦ The 14-item version of this measure will be used to capture ‘persecutory beliefs’ and ‘benevolent beliefs’.

11. Secondary (clinical) outcome: Revised Green Paranoid Thoughts Scale (R-GPTS) [35]
  ◦ Interventions that target one specific symptom of psychosis may have an impact upon other psychosis symptoms. This 18-item measure will capture any impact upon paranoid delusions.

#### Health economic measures

12. Client Service Receipt Inventory (CSRI-UK) [29]
  ◦ This instrument will be used to collect information on service utilisation, income, accommodation and other cost-related variables
  ◦ Its primary purpose is to allow resource-use patterns to be described and support costs to be estimated.

13. EQ-5D-5L [36]
  ◦ This standardised instrument will be used as a measure of health-related quality of life relevant to a wide range of health conditions and treatments
  ◦ The descriptive system comprises five dimensions: mobility, self-care, usual activities, pain/discomfort and anxiety/depression. Each dimension has 5 levels: no problems, slight problems, moderate problems, severe problems and extreme problems.

14. SF-12 v2 [37]
  ◦ This generic health survey captures information about functional health and well-being from the patient’s point of view.
  ◦ It consists of 12 questions that measure 8 health-related domains around physical and mental health. The physical health-related domain covers General Health, Physical Functioning, Role-Physical and Body Pain while the mental health-related domain covers Vitality, Social Functioning, Role Emotional and Mental Health.

### Participant timeline {13}

**Fig. 1:**
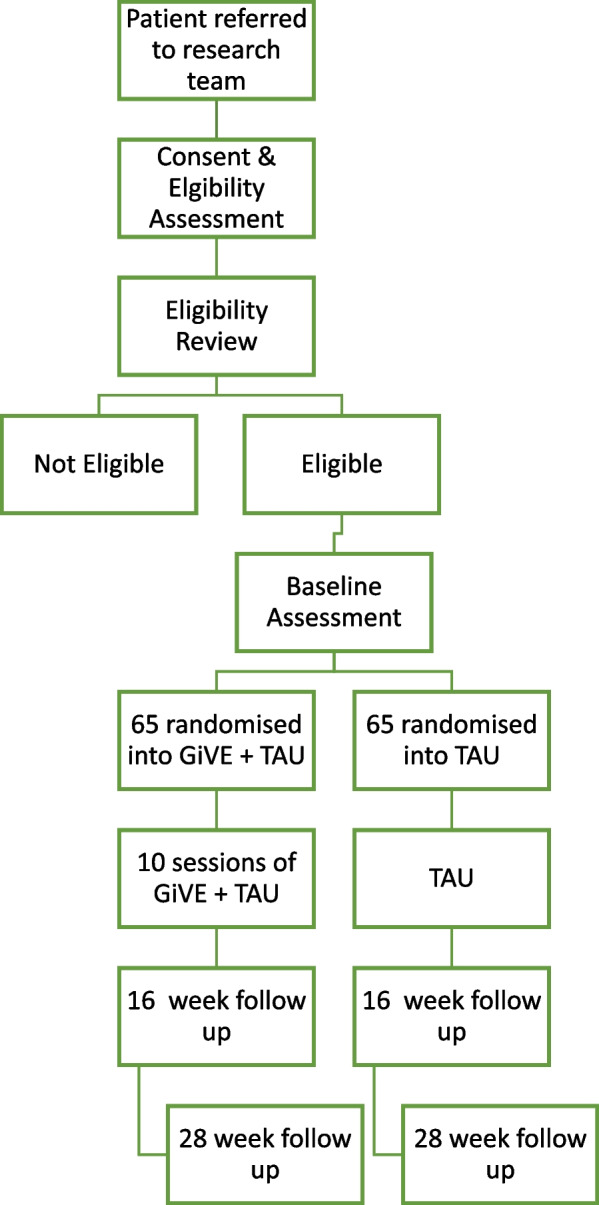
Trial/recruitment flowchart

Figure 1 illustrates the trial/recruitment flowchart.

### Sample size {14}

Under simple randomisation, to detect a medium effect size of Cohen’s *d* = 0.5 with 90% power for 5% significance with one baseline measure and two follow-up measures, with correlation between these of 0.6 [38], 37 participants per arm are required. The adjusted standard deviation obtained from this calculation is 0.663 (step 1).

To estimate the power for a design with clustering in one arm only, we used the following parameters: 6 therapists each with a caseload of 8 participants completing follow-up assessments, an intracluster correlation coefficient (ICC) of 0.05 [39], an equally sized group (for maximum efficiency) of 6 × 8 = 48 unclustered participants for the treatment as usual arm (ICC = 0), a 5% significance level, adjusted variance from Step 1, i.e. 0.663, a medium effect size of Cohen’s *d* = 0.5. This resulted in an estimated power of 88%.

We assume 26% attrition so the sample size to be recruited is 96/(1 − 0.26) = 130 participants, i.e. 65 per group implying a caseload of between 10 and 11 participants per therapist over the course of the intervention.

### Recruitment {15}

Participants will be recruited through referrals from the Care Coordinators (CC) of psychosis patients (or other appropriate members of the clinical team) in the three sites. CCs will be given a Participant Information Sheet (PIS) and referral forms when first approached about the study by an RA. CCs will be asked to share the PIS during face-to-face and remote consultations with any patients who are potentially eligible for the study. If a patient shows an interest in the study and gives their verbal agreement to be contacted by an RA, the CC will be asked to facilitate the completion of a referral form. Once a referral has been received by the research team, the potential participant will be contacted by the RA to discuss the study further and arrange a consent and eligibility meeting. The potential participant will have a copy of the PIS at least 24 h before the consent and eligibility meeting, so they will have time to read the information, discuss it with friends and family, and formulate any questions they may have.

The consent and eligibility meeting will be completed face-to-face, unless a patient expresses a preference for a remote meeting. At this meeting, potential participants will read through the PIS with the RA to ensure they have understood the study and can ask questions. Potential participants will be invited to consent to take part in the study by reading and signing three copies of the consent form: one for the research study to be filed in the study file, one for the participant to keep and one for the participant’s care team to be emailed to the Care Coordinator and uploaded to the electronic health records. If the meeting is conducted remotely, the patient will consent verbally, and three copies of the consent form will be completed and distributed by the RA.

Eligibility data will be entered into an online platform (called REDCap) during the meeting and be accessible by the Brighton & Sussex Clinical Trials Unit (CTU). Patients will be offered reimbursement of £20 for attending the consent and eligibility meeting and travel expenses will be made available.

An additional recruitment strategy at the Sussex site will involve the use of the Everyone Counts scheme. SPFT has the Everyone Counts scheme in place for consent to contact patients about research opportunities directly. The Everyone Counts scheme is viewed as being a task in the public interest and as such has been approved by the SPFT Trust Board after consultation with the Information Governance Lead. Members of the Research & Development Department will contact potentially eligible patients to discuss the study and invite them to take part. The report of potential participants is only accessible to a limited number of authorised individuals in the R&D Department. Interested patients will be able to contact either the R&D Department or the research team directly and enquire about the study. They will be invited to discuss the study with their CC who can then complete a referral form. The research team could support potential participants to discuss the study with their CC if this is needed.

An additional strategy will be used in PCFT, whereby individuals who have participated in other trials within the Trust and have given consent to be contacted about future studies may be contacted.

## Assignment of interventions: allocation

### Sequence generation {16a} and Concealment mechanism {16b}

The CTU will set up the online randomisation process. The RAs, the Trial Statistician and the CTU Statistician will be blind to participant allocation. Following the baseline assessment, participants will be randomised, stratified by site, by the TM in permuted blocks of randomly varying length using the online randomisation module of REDCap and allocated to one of the trial arms. The next allocation is only revealed upon request. Further, the permuted blocks make the next allocation almost impossible to guess.

### Implementation {16c}

The TM will communicate the allocation to the appropriate unblinded members of the research team. An unblinded member of the research team will then contact the participant by telephone to inform them of their group allocation. APs will be notified by the TM about the participants who are allocated to the GiVE arm and will be asked to arrange a first appointment, if possible, within two weeks following the randomisation.

## Assignment of interventions: Blinding

### Who will be blinded? {17a}

The CTU will set up the online randomisation process. The RAs, the Trial Statistician and the CTU Statistician will be blind to participant allocation. Measures to maintain blinding will include: (1) participants being reminded at the beginning of each assessment interview to not disclose the arm to which they have been allocated; (2) blinded members of the research team being shielded from discussion of participants in forums where the possibility of determining participant allocation could occur; (3) researcher access to electronic health records being restricted; and (4) consideration given to office allocation and all administrative processes of blinded-vs-unblinded members of the research team. ‘Blind’ awareness and education will be promoted throughout the study. To test the success of blinding, the RA who assesses each participant will be asked to guess the allocation group for the participant at the end of the final assessment. Reported breaks in blinding will be recorded.

### Procedure for unblinding if needed {17b}

If unblinding occurs at the beginning of an assessment, the assessment will be re-blinded by re-allocating a ‘blind’ RA to collect and score study data wherever possible.

## Data collection and management

### Plans for assessment and collection of outcomes {18a}

**Fig. 2:**
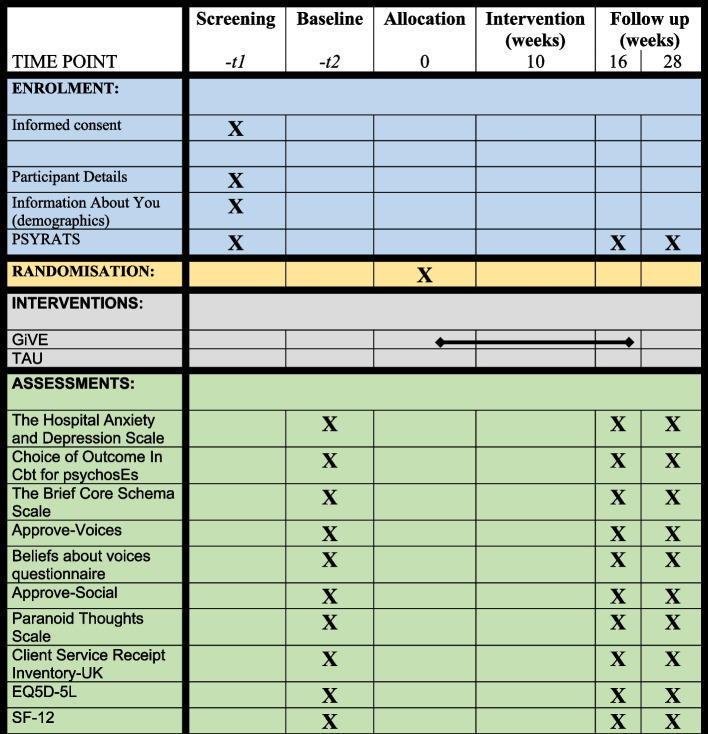
Details of assessments at each visit

See Fig. 2 for details of assessment at each visit.

### Screening measures

The assessment of eligibility in relation to the inclusion and exclusion criteria will be supported by the use of the Psychotic Symptoms Rating Scale (PSYRATS-AH) [21] (described below).

### Clinical measures—primary

Psychotic Symptoms Rating Scale (PSYRATS-AH) [21] Distress Scale – PSYRATS-AH is an 11-item rating scale designed to measure the severity of different dimensions of the voice hearing experience. Items are grouped together in four factors [40]; distress (negative content, distress, and control), frequency (frequency, duration, and disruption), attribution (location and origin of voices), and loudness (loudness item only). The ‘distress’ subscale measures the impact that voices have on the individual. The measure has established psychometric properties. The primary outcome will be measured at 16 weeks post-randomisation.

### Clinical measures—secondary

Secondary outcomes will evaluate: 1) the characteristics of voice hearing experiences (PSYRATS subscales of attribution [2 items], loudness [1 item] and frequency [3 items]); 2) mental health problems commonly experienced by people with psychosis (anxiety and depression [Hospital Anxiety and Depression Scale] [30] and paranoia [Paranoid Thoughts Scale] [35]): 3) variables that have been associated with the impact of voice hearing (negative beliefs about the self [Self Scale of the Brief Core Schema Scale] [32], negative beliefs about voices [Beliefs About Voices Questionnaire – Revised] [34], and negative relating to voices [Approve -Voices] [34] and other people [Approve-Social] [33]; and 4) personal and social recovery-oriented outcomes (goals for the outcome of CBTp [CHOICE – short form] [31].

### Health economic measures

#### EQ5D-5L [36]

EQ5D-5L [36] is a standardised instrument used as a measure of health-related quality of life that can be used in a wide range of health conditions and treatments. The descriptive system comprises five dimensions: mobility, self-care, usual activities, pain/discomfort and anxiety/depression. Each dimension has five levels: no problems, slight problems, moderate problems, severe problems and extreme problems.

#### Client Service Receipt Inventory (CSRI-UK) [29]

Client Service Receipt Inventory (CSRI-UK) [29] is a well validated adaptable research instrument used in mental health settings to collect information on health, social and voluntary service utilisation, informal care, accommodation, other public services (e.g. police) and private out-of-pocket expenses incurred. Its primary purpose is to allow resource-use patterns to be described and costs to be estimated.

#### Short Form 12 (SF12) [3]

This generic health survey captures information about functional health and well-being from the patient’s point of view.

### Plans to promote participant retention and complete follow-up {18b}

Efforts will be made to engage all participants in follow-up assessments. RAs will flexibly engage patients, offering appointments at times and locations which best suit participants and offering shorter and split assessment sessions as needed. Participants will be offered reimbursement of £20 per assessment point (i.e., eligibility, baseline, 16 and 28 weeks) and travel expenses will be made available. Retention rates will be monitored by the TM at least weekly and the research team on a monthly basis throughout the trial. The Lived Experience Advisory Panel (LEAP) will be asked to provide consultation regarding retention rates as part of their oversight role.

### Data management {19}

The research team adhere to the good practice, standards and principles which are set out in the Sussex Partnership Policy for Data Protection, Security and Confidentiality. This policy reflects the recommendations from current legislation, including The Caldicott Report (1997), the British Standard (ISO IEC 27002) for Information Security, the General Data Protection Regulation 2018 and the Sussex Partnership NHS Foundation Trust Research Policy 2021.

Data management and analysis will be in accordance with CTU Standard Operating Procedures (SOPs). Processes to promote data quality will be guided by the CTU Data Management Plans (available upon request from the first author).

All research will be carried out under the above standards and will be reviewed by an NHS Ethics Committee, the Health Research Authority and the respective R&D Department under the UK Framework for Health and Social Care Research (2017).

All members of the research team and any other individuals from collaborating Trusts or Universities involved in collecting, inputting, processing, using and sharing data will have had Information Governance Training.

The management of the data will be a standing item on the agenda of the monthly meeting of the research team.

### Confidentiality {27}

Consent forms will be stored in locked filing cabinets on NHS premises.

Participants’ names will be included on consent forms during the eligibility assessment. Following this, the RA will provide each participant with a unique study identification code. The participant’s name and unique study identification will be separated. A separate participant identification log linking participants’ names and personal details to the unique study identification codes will be kept securely at each site; this file will be kept in a password-protected file on an NHS trust computer that only members of the research team will have access to.

Quantitative data collected using REDCap will be pseudonymised by using the unique study identification codes. REDCap data is stored securely on the University of Sussex servers and is regularly backed up. Access to REDCap will be limited to the research team.

The adherence data will be collected using an audio recording device. Once the data has been collected, it will be stored on a computer that is password-protected, within a password-protected file and deleted from the audio recording device. The transcripts of the interviews will also be kept in password-protected files. The transcripts will not contain any identifiable information.

All electronically stored data (i.e., REDCap file with questionnaire scores and audio files) will be anonymised, and kept in encrypted files, on a shared drive, with access to the shared drive via username and password. Only members of the research team will have usernames and know the password and will therefore be able to access the electronic data.

All paper records of research data will be archived and then destroyed after 10 years.

All personal information will be destroyed when it is no longer needed.

### Plans for collection, laboratory evaluation and storage of biological specimens for genetic or molecular analysis in this trial/future use {33}

N/A There will be no biological specimens collected.

## Statistical methods

### Statistical methods for primary and secondary outcomes {20a}

Participant flow through the trial will be shown in a chart according to the CONSORT Statement 2010. Baseline sociodemographic characteristics and outcomes will be summarised by trial arm using descriptive statistics appropriate to distribution. The primary analysis will be conducted using a mixed effects model with clustering by therapist in the intervention group only and control participants forming their own clusters of size 1. We will include fixed effects for PSYRATS-AH Distress at baseline, the stratification variable (site) and allocation and a random effect for therapist/control participant and a random effect to account for repeated observations within participants. A small sample correction (e.g. Satterthwaite’s) will be applied due to the small number of therapists. We will report the adjusted difference in mean PSYRATS-AH Distress between arms, its 95% confidence interval and p value at each time point. Statistical significance is set at the 5% level.

Secondary outcomes will be analysed using mixed effects models appropriate to outcome distribution controlling for baseline voice hearing distress (PSYRATS-AH). A detailed statistical analysis plan will be written by the CTU Statistician and Trial Statistician, agreed with the research team and reviewed by the Trial Steering Committee (TSC).

### Interim analyses {21b}

N/A. There are no planned interim analyses.

### Methods for additional analyses (e.g. subgroup analyses) {20b}

#### Health Economic analysis

The resource requirements of each arm (GiVE with TAU vs. TAU alone) will be investigated using trial details on the delivery of GiVE and data gathered from participants for TAU. Staff time will be costed using national tariffs, inclusive of oncosts and overheads, [41] materials used in the intervention will be valued at cost to the study. Participants will be asked to complete the Client Service Receipt Inventory (CSRI [29]) at baseline, at the end of the intervention (16 weeks) and at the 28-week follow-up to record primary, secondary and community-based health care, including activity classed as standard care. The CSRI is a well validated instrument for use in mental health settings, that can be customised to individual projects, and which includes recording of social, criminal justice and voluntary sector input, informal caring and private out-of-pocket expenditures. Service use will be costed using national tariffs [41]. Variability in standard care will be explored. Differences in service use between groups will be investigated for indications of potential savings associated with GiVE that might offset the intervention costs.

Health-related quality of life (HRQoL), the primary outcome for the economic evaluation, will be recorded at all three assessment points (baseline, 16 and 28 weeks) using both EQ-5D-5L [36] and SF-12 v2 [37] for calculation of QALYs as the latter may be more sensitive to changes in the psychological status. Variability in findings from EQ-5D-5L and SF-12 v2 will be compared.

The full range of outcomes will be investigated in a cost consequences framework and a cost-effectiveness analysis will be conducted. Group mean costs and QALYs will be analysed in line with other outcomes using mixed effects linear regression, taking account of key baseline prognostic factors and clustering, and with QALYs adjusted for baseline utility scores. Cost-effectiveness will be expressed as incremental cost-effectiveness ratios in a fully incremental analysis. In addition, Incremental Net Monetary Benefit at various cost-effectiveness thresholds, accompanied by Cost-Effectiveness Acceptability Curves will be presented. If necessary, nonparametric bootstrapping techniques will be used to characterise uncertainty in skewed outcomes. Cost-effectiveness will also be calculated in terms of cost per unit improvement in the PSYRATS-AH Distress scale. Data gathered during the study will form a basis for potential subsequent economic modelling.

#### Adherence and competence analysis

Therapeutic drift will be minimised by the structure and detail of the workbook and close supervision of the APs by the Site Leads. Adherence to the workbook will be assessed through APs completing a session checklist at the conclusion of each session. All therapy sessions will be audio-recorded (with the participant’s consent – which will be verbally re-affirmed at the beginning of each therapy session) and a random 10% sample rated for adherence and competence by an independent CBT expert. These recordings will be rated for adherence using the session checklist. In addition to providing an independent assessment of the AP’s delivery of the session content, cross-referencing of the self-report and independently rated checklists will offer insights about the ability of APs to monitor their own performance. Session recordings will also be rated for competence using a version of the Cognitive Therapy Scale-Revised (CTS-R) [28] that has been adapted for use within the training of ‘lower intensity’ therapists within the Improving Access to Psychological Therapies services within Sussex.

#### Development of the model for future implementation

The development of an implementation model will use a Normalisation Process Theory framework [42] and build on the key findings from the process evaluation conducted within the feasibility RCT. Specifically, referring clinicians proposed that the GiVE intervention should be embedded into routine service, and be clearly positioned as a CBT-informed intervention, delivered in close collaboration with both CCs (who could further consolidate and support learning after the completion of the GiVE intervention) and highly trained therapists (who could offer subsequent CBTp, where needed). With this in mind, the development of the implementation model will involve two components. Initially, over a six-month period, a series of stakeholder group consultations will be conducted with policy makers, clinicians and highly trained therapists. We will aim to recruit 8–15 stakeholders including at least 2 each of policy makers, CC referrers and expert therapists. Policy makers will be recruited from the national advisory groups involved in developing new psychological therapy roles. CCs and expert therapy leads will be recruited from study sites. Participants will meet either in person or online. Participants will be invited to take part in an interview where they will review the model and consider approaches to implementation in terms of: coherence with current provision; cognitive participation; collective action; and reflexive monitoring. Interviews will be facilitated by either one (individual) or two (small-group) researchers and guided by a topic-guide based on NPT theory. They will be audio-recorded and recordings saved in a manner that is consistent with study and Trust protocols. Audio recordings will be transferred as soon as possible to secure NHS or University servers. Once transcribed, audio-recorded interviews will be deleted. Data will be analysed thematically using a thematic framework analysis approach [43].

Analysis of the transcripts from the consultations will produce a list of potential components of the model and considerations for implementation. A subsequent Delphi consultation, conducted over three months, will be implemented with these same consultants to reach consensus, especially where there were differences of opinion on the core components of the model derived from the qualitative findings, on which there is a group consensus. An online questionnaire format will elicit ratings of the feasibility and importance, and qualitative feedback on key components of the model. A second round will share key learning from the first round to encourage consensus on the final model. The model will be written-up during the final three months of the proposed study.

#### Evaluation of use and experience of self-help materials

A qualitative evaluation will aim to identify barriers and facilitators that may exist for participants when making use of the self-help resources that comprise the GiVE intervention, namely the CHOICES app, the self-help book [21] and the workbook [22]. An increasing number of psychological interventions for symptoms of psychosis are making use of ‘Therapeutic Resources’ (equipment that facilitates a psychological intervention partially or wholly independent of contact from a therapist). The CHOICES app, the self-help book and workbook are examples of Therapeutic Resources (TR). It is therefore important to know what might help and hinder engagement in using TR such as those within the GiVE intervention. Enablers and barriers to engagement with TR have been identified in other trials (digital TR [44, 45]), and also within the GiVE series of trials (paper-based TR [46]). However, there has been no attempt to explore the experiences of participants when they are offered a suite of both digital and paper-based TRs. This leaves important gaps in knowledge when considering the training and supervision of therapists who are delivering interventions that make use of TR as a core part of the intervention, and the implementation of guided self-help interventions in a psychosis population.

This evaluation will aim to explore the experiences via an opportunity sample of 15 participants who: 1) are allocated to the GiVE intervention arm of the trial; 2) have given consent to be contacted for further research when initially consenting to participate within the trial; and 3) have completed the 28-week assessment. Eligible participants will be identified by the TM and invited to participate in the evaluation by an RA. Eligible participants expressing interest will be contacted by a researcher to obtain informed consent to be part of the qualitative evaluation. After participants have consented, arrangements will be made for an interview to be conducted by telephone or video call. The interview will be guided by a semi-structured interview schedule and transcribed verbatim. Qualitative data will be analysed using Thematic Analysis [47]. All qualitative data analysis will be undertaken using NVivo, a qualitative data analysis software. Participants will be reimbursed £20 in Amazon Vouchers for their contributing to the interview.

### Methods in analysis to handle protocol non-adherence and any statistical methods to handle missing data {20c}

Analysis will follow intention-to-treat principles and missing data may be multiply imputed if the missing data mechanism is considered to be Missing at Random (MAR). We will also conduct a complier average causal effect analysis of the primary outcome.

### Plans to give access to the full protocol, participant level data and statistical code {31c}

An anonymised dataset will be deposited within the University of Sussex Research Repository to facilitate open access for other researchers. The reuse and sharing of anonymised data will be made explicit to participants on the study consent form. Identifiable information will not be shared with anyone outside of the research team.

## Oversight and monitoring

### Composition of the trial steering committee {5d}

A Trial Steering Committee (TSC) will be established according to MRC guidelines [48] and will be chaired by a senior clinical academic. Membership will include at least two independent experts and two independent lay members. The TSC will meet at least annually and will provide overall supervision of the trial, monitoring adherence to the protocol and providing independent advice on all aspects of the trial.

A separate Lived Experience Advisory Panel (LEAP) will provide Patient and Public Involvement (PPI) oversight of the trial. LEAP members will receive training to facilitate their involvement and meet regularly during the course of the study to consult on issues concerning informed consent and participant well-being, development of accessible study materials, recruitment and retention, and the interpretation of findings and dissemination. An ‘involvement log’ will track the influence of the advice offered to the Research Team by the LEAP.

### Composition of the data monitoring committee, its role and reporting structure {21a}

A Data Monitoring & Ethics Committee (DMEC) will meet at least annually and report to the TSC. It will have access to all unblinded trial data and will receive regular reports on adverse events. Membership of the DMEC will be independent of the applicants and of the TSC. An independent senior statistician will be appointed as chair and the group will also comprise independent senior clinical academics. The CI, trial coordinator and the trial statistician will attend parts of the DMEC meeting to provide reports but will not be members of the DMEC.

### Adverse event reporting and harms {22}

Any unfavourable and unintended sign, symptom or illness that develops or worsens during the period of the study will be classified as an adverse event (AE), whether or not it is considered to be related to the study treatment. Adverse events will include: an exacerbation of a pre-existing illness; an increase in the frequency or intensity of a pre-existing episodic event or condition requiring additional contact from care teams; a condition that is detected after trial intervention administration; and continuous persistent disease or a symptom present at baseline that worsens following administration of the trial treatment—and may be expected or unexpected. Serious Adverse Events (SAEs) are those considered to be life-threatening, resulting in death, requiring inpatient hospitalisation or prolongation of existing hospitalisation, resulting in significant or persistent incapacity/disability or a birth defect or congenital abnormality. The number (events and individuals) and nature of all events (AE and SAE) reported to blind and unblind members of the research team will be recorded.

The period for adverse event reporting will be following the signing of the study consent form until last follow-up assessment 28 weeks after randomisation. All AEs will be recorded and reviewed by a Site Lead. If an AE is considered to be serious (an SAE), it will be reviewed for causality and expectedness by the Chief Investigator and an independent rater. The TSC will be informed of the number, nature and review outcome for all SAEs and will be asked to recommend any necessary actions. SAEs will be reported to the Sponsor, DMEC and NHS Research Ethics Committee as appropriate.

### Frequency and plans for auditing trial conduct {23}

The roles of the TSC and the DMEC are to ensure the trial is conducted to a high standard; these committees are independent from the investigators and sponsor.

### Plans for communicating important protocol amendments to relevant parties (e.g. trial participants, ethical committees) {25}

Any protocol amendments will be submitted for approval to the Research Ethics Committee and Health Research Authority; subsequent changes will be recorded in the study protocol and the ISRTCN registration.

### Dissemination plans {31a}

Trial findings will be disseminated in scientific publications, including efficacy outcomes and learning from the development of the implementation model. Findings will be disseminated to participants’ and patient organisations. LEAP members will participate in dissemination including use of social media to disseminate findings, producing leaflets for wide distribution and submitting a summary of findings to a non-academic journal. Findings will be presented at patient events and at local, national and international conferences.

## Discussion

CBTp is an evidence-based psychological therapy recommended for psychosis patients within the UK. However, only a minority of patients are offered CBTp. Limited resources have been cited as a prominent reason for this lack of access, leading to calls for shorter forms of CBTp to be developed that can be delivered by briefly trained therapists.

We have responded to this call by developing a brief and targeted form of CBTp for distressing voices – Guided, self-help intervention for voices (‘GiVE’). Findings from a feasibility RCT suggested that evaluation of the GiVE intervention is feasible when the intervention is delivered by briefly trained therapists (in the form of APs). The current study will offer evidence concerning the clinical and cost-effectiveness of the GiVE intervention when delivered to psychosis patients by APs.

CBTp offered in less resource-intensive forms has the potential to generate benefits for: 1) individual patients (reduced distress and enhanced recovery and enhanced quality of life); 2) service-level patient benefit (increased access to evidence-based psychological therapies); and 3) economic benefits to the NHS (in terms of the reduced use of mental health inpatient services).

### Trial status

Recruitment to the trial commenced in October 2022. At present, recruitment and data collection will continue until April 2024.

## Data Availability

An anonymised dataset will be deposited within the University of Sussex Research Repository to facilitate open access for other researchers. The reuse and sharing of anonymised data will be made explicit to participants on the study consent form. Identifiable information will not be shared with anyone outside of the research team. The self-help materials that support the delivery of the GiVE intervention are already in the public domain.

## Abbreviations

AP: Assistant psychologists
CBTp: Cognitive behaviour therapy for psychosis
CC: Care Coordinator
CHOICE: CHoice of Outcome In Cbt for psychoses
CSRI: Client Service Receipt Inventory
CTU: Clinical Trials Unit
DMEC: Data Monitoring & Ethics Committee
EBE: Experts by Experience
GiVE: Guided self-help CBT intervention for voices
HRQoL: Health-related quality of life
MRC: Medical Research Council
NICE: National Institute for Health and Care Excellence
NHS: National Health Service
NIHR: National Institute for Health and Care Research
LEAP: Lived Experience Advisory Panel
PIS: Participant Information Sheet
QALY: Quality-Adjusted Life Years
PSYRATS: Psychotic Symptoms Rating Scale
RA: Research Assistant
RCT: Randomised controlled trial
SF12: Short Form 12
TAU: Treatment as usual
TSC: Trial Steering Committee
